# Upregulation of miR-129-5p increases the sensitivity to Taxol through inhibiting HMGB1-mediated cell autophagy in breast cancer MCF-7 cells

**DOI:** 10.1590/1414-431X20198657

**Published:** 2019-10-28

**Authors:** Ying Shi, Weihua Gong, Lu Lu, Yunfeng Wang, Jingjing Ren

**Affiliations:** 1 Clinical Laboratory, The Third Affiliated Hospital of Zhengzhou University, Zhengzhou, China; 2 Clinical Laboratory, Beijing Chaoyang Emergency Medical Center, Beijing, China; 3 Clinical Laboratory, Xiang Cheng City First Person Hospital, Zhoukou, China

**Keywords:** miR-129-5p, HMGB1, Autophagy, Taxol, Breast cancer

## Abstract

Although Taxol has improved the survival of cancer patients as a first-line chemotherapeutic agent, an increasing number of patients develop resistance to Taxol after prolonged treatment. The potential mechanisms of cancer cell resistance to Taxol are not completely clear. It has been reported that microRNAs (miRNAs) are involved in regulating the sensitivity of cancer cells to various chemotherapeutic agents. In this study, we aimed to explore the role of miR-129-5p in regulating the sensitivity of breast cancer cells to Taxol. Cell apoptosis and autophagy, and the sensitivity of MCF-7 cells to Taxol were assessed with a series of in vitro assays. Our results showed that the inhibition of autophagy increased the Taxol-induced apoptosis and the sensitivity of MCF-7 cells to Taxol. Up-regulation of miR-129-5p also inhibited autophagy and induced apoptosis. Furthermore, miR-129-5p overexpression increased the sensitivity of MCF-7 cells to Taxol. High mobility group box 1 (HMGB1), a target gene of miR-129-5p and a regulator of autophagy, was negatively regulated by miR-129-5p. We found that interference of HMGB1 enhanced the chemosensitivity of Taxol by inhibiting autophagy and inducing apoptosis in MCF-7 cells. Taken together, our findings suggested that miR-129-5p increased the chemosensitivity of MCF-7 cells to Taxol through suppressing autophagy and enhancing apoptosis by inhibiting HMGB1. Using miR-129-5p/HMGB1/autophagy-based therapeutic strategies may be a potential treatment for overcoming Taxol resistance in breast cancer.

## Introduction

Breast cancer is the most common cancer-related cause of death in females worldwide (1). Although surgery and radiotherapy can be used for the treatment of breast cancer, chemotherapy is still common and essential and can reduce clinical stage, kill or reduce micrometastases, and prevent distant metastases (2,3). Taxol is among the most widely used chemotherapy agents in the treatment of breast cancer (4). It stabilizes microtubules, leading to cell cycle arrest and, ultimately, cell death (5–7). Nevertheless, intrinsic and acquired drug resistance to Taxol remains an important factor limiting the clinical efficacy (8,9). Thus, it is important to enhance the sensitivity of breast cancer to Taxol. However, the potential mechanisms for the development of Taxol resistance are still unclear.

Micro RNAs (miRNAs) are a class of short, endogenous, non-coding RNAs (∼20–24 nucleotides) that regulate the expression of a diversity of genes (10). They have been identified to be involved in a variety of biological processes (11–13). Recently, an increasing number of studies have reported that dysregulation of miRNAs might be related to drug resistance (14–16). With the deepening of miRNA research, targeting their downstream genes to regulate multidrug resistance has become a research hotspot. MiR-129-5p, a tumor inhibitor, has been validated to inhibit cancer cells migration, proliferation, differentiation, and epithelial-mesenchymal transition (17–20). In addition, miR-129-5p-mediated beclin-1 suppression inhibits endothelial cell autophagy in atherosclerosis (21). With the in-depth study on the relationship between miRNAs and cancer resistance (22), more and more researchers consider that miR-129-5p is an important molecule involved in cancer resistance (23). Regulating the expression of miR-129-5p might be a potential strategy for the treatment of cancer resistance.

Autophagy is a well-known important protective mechanism that can help cells against adverse growth and promote cell survival (24,25). Protective autophagy can resist chemotherapeutic-induced cell apoptosis and cell injury in the course of anti-tumor treatment (26,27). High mobility group box 1 (HMGB1) is a regulator of autophagy as a ubiquitous nuclear protein, which can regulate and promote various DNA-related activities such as transcription, replication, and repair (28–30). Recently, studies indicated that HMGB1 plays an essential role in chemotherapy sensitivity through modulating protective autophagy in a number of cancers, including breast cancer (31,32). Thus, targeting HMGB1-mediated autophagy might improve the chemosensitivity of cancer cells. Luo et al. (33) reported that miR-129-5p regulated autophagy by targeting HMGB1 in breast cancer MCF-7 cells. However, no study has focused on the association of miR-129-5p dysregulation and the sensitivity of breast cancer cells to Taxol by targeting HMGB1.

## Material and Methods

### Cell culture and drug treatment

MCF-7 cells were purchased from the American Type Culture Collection (ATCC, USA). MCF-7 cells were maintained in RPMI-1640 (Solarbio, China) medium containing 10% fetal bovine serum at 37°C and 5% CO_2_. MCF-7 cells were cultured two days before treatments. Cells were treated with 3-methyladenine (3-MA; Selleck, USA) an autophagy inhibitor, for 2 h before Taxol (Xi’an Haoxuan Bio-tech Co., Ltd., China) treatment for 24 h.

### Cell proliferation assay

MCF-7 cells were seeded onto 96-well cell culture cluster plates at a density of 8×10^3^ cells/well in 100 μL RPMI 1640 medium (Solarbio, China) supplemented with 10% fetal bovine serum (Gibco BRL, USA) and grown overnight. After treatment with different concentrations (3.9, 7.8, 15.6, 31.2, 125.0, 500.0, and 1000.0 nM) of Taxol for 24 h, cell proliferation was measured using the Cell Counting Kit-8 (Dojindo, China) according to the manufacturer's protocol. The results are reported as a ratio of cell proliferation/inhibition and the experiments were repeated at least three times. Inhibition of proliferation rate (%) = (1 − Ab (absorbance) value of experimental group / Ab value of control group) × 100%.

### miRNA and siRNA transfection

MiR-129-5p mimics (miR-129-5p), miRNA control (miR-NC), small-interfering RNA HMGB1 (si-HMGB1), and small-interfering RNA control (si-NC) were purchased from Sangon Biotech (China). They were transfected with LipoHigh reagent (Sangon Biotech) into MCF-7 cells according to the manufacturer's instructions. Cell lysates were prepared for subsequent experiments after transfection.

Total RNA isolation from MCF-7 cells was performed using Trizol (Sangon Biotech). cDNA was synthesized using Revert Acc kit (Sangon Biotech) according to the manufacturer's instructions. Quantitative real-time PCR was performed on an ABI 7500 real-time system (Applied Biosystems, USA) according to the manufacturer's protocol. Data were calculated using the 2^-ΔΔCT^ method based on the internal control. The relative expression of miR-129-5p was normalized to U6 expression. The relative expression of HMGB1 mRNA was normalized to GAPDH expression. The data set was generated by at least three independent experiments. PCR primers are shown in Table 1.

**Table 1:** Polymerase chain reaction primers.

Name	Forward primer	Reverse primer
miR-129-5p	5′-CTTTTTGCGGTCTGGGCTTG-3′	5′-AACGCTTCACGAATTTGCGT-3′
U6	5′-CTCGCTTCGGCAGCACA-3′	5′-AACGCTTCACGAATTTGCGT-3′
HMGB1	5′-ACAAGGCCCGTTATGAAAGA-3′	5′-GAAGAGGAAGAAGGCCGAAG-3′
GAPDH	5′-CAGGAGGCATTGCTGATGAT-3′	5′-GAAGGCTGGGGCTCATTT-3′

### Western blotting analysis

MCF-7 cells were lysed with RIPA lysis buffer (Solarbio), and western blotting was performed using standard procedures. The concentration of protein was determined using the BCA protein quantitation kit (Sangon Biotech). Equal quantities of denatured protein samples were resolved on 12% SDS-polyacrylamide gels based on the targeted molecular weight and then transferred onto PVDF membranes (Millipore, USA). Membranes were incubated with a specific primary antibody against HMGB1 (1:10000 dilution; Abcam, USA), p62 (1:10000 dilution; Abcam), and LC3B (1:1000 dilution; Cell Signaling Technology, USA). Proteins were visualized using ECL system (Millipore). GAPDH was used as an internal control.

### Flow cytometry for apoptosis

MCF-7 cells were seeded overnight in six-well plates. Cells were transfected with siRNA-HMGB1, miR-129-5p, or control for 24 h and then treated with IC_30_ Taxol. After 24 h, cells were harvested and stained with annexin V-FITC and propidium iodide (PI) following the manufacturer’s protocol (BD Biosciences, USA). Finally, stained cells were analyzed by flow cytometry (BD FACSCanto, BD Biosciences). Three biological experiments were performed.

### Immunofluorescence

First, MCF-7 cells were seeded onto cover-slips and then fixed in 4% paraformaldehyde for 30 min at room temperature. Cells were washed with PBS three times, blocked with 5% goat serum (Ding Guo Chang Sheng, China), and permeabilized with 0.3% Triton X-100 for 15 min. Cells were incubated with rabbit polyclonal anti-HMGB1 (1:200 dilution, Abcam) at 4°C overnight. After washing in PBS, cells were incubated with FITC-labeled anti-rabbit secondary antibody (Ding Guo Chang Sheng) for 1 h at room temperature. Images were acquired using a fluorescence microscope (Olympus, Japan).

### Bioinformatics analysis

Overlapping target genes were predicted using miRDB (mirdb.org), microRNA.org, and TargetScan (targetscan.org). The punitive binding sites among miR-129-5p and HMGB1 were predicted using TargetScan. The expression of HMGB1 in normal breast tissue and breast cancer tissue were searched in the online databases Oncomine (https://www.oncomine.org/resource/login.html) and the Human Protein Atlas (https://www.proteinatlas.org/).

### Statistical analysis

Data were analyzed using SPSS 21.0 statistical software (IBM, USA). All results are reported as means±SD. Student's *t*-test or one-way ANOVA were used to analyze the differences between treatment groups. P<0.05 was considered statistically significant.

## Results

### The sensitivity of MCF-7 cells to Taxol was increased by inhibiting autophagy and inducing apoptosis

**Figure 1.**
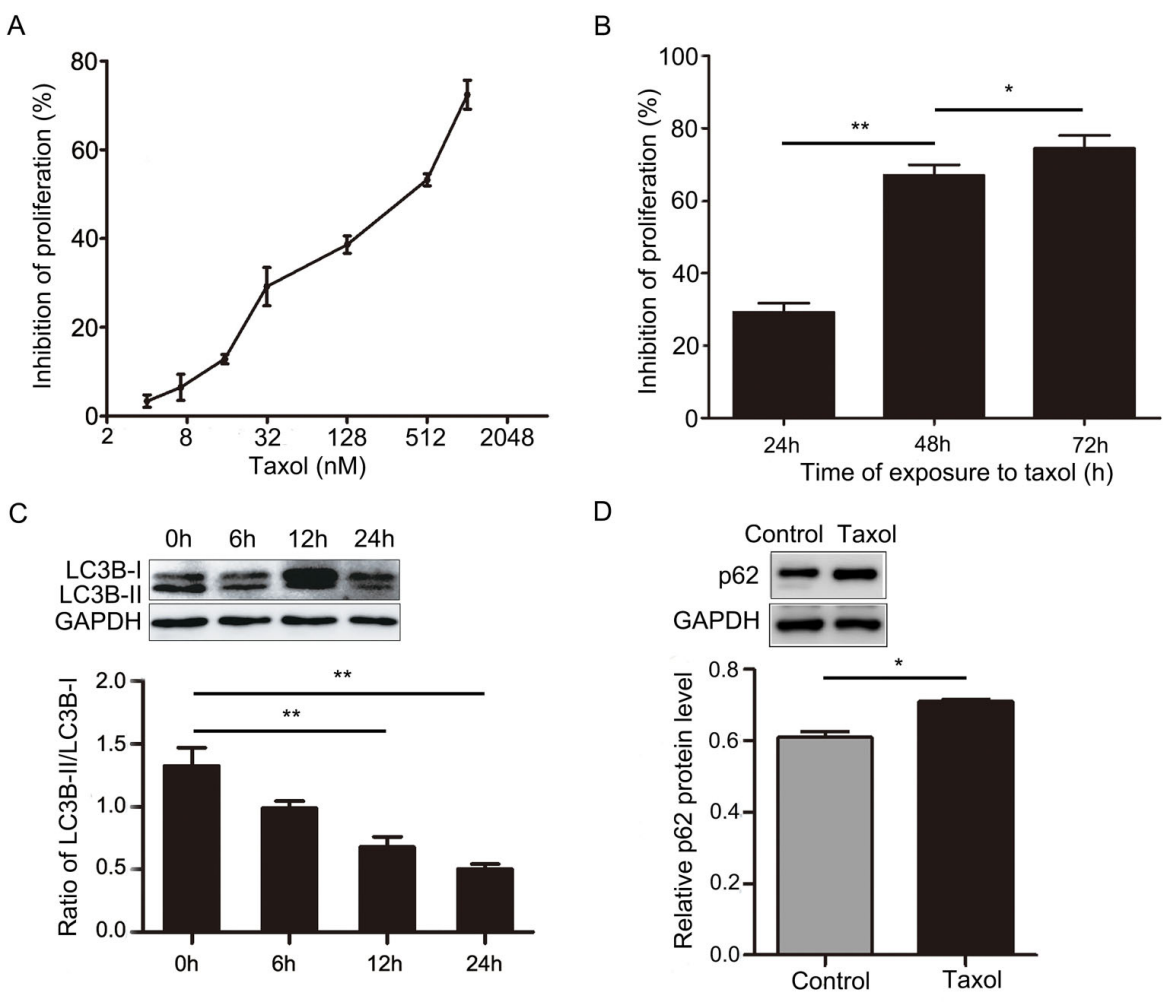
**A**, Cell proliferation was determined by the CCK-8 assay after treatment with different concentrations of Taxol for 24 h. **B**, Cell proliferation was determined by the CCK-8 assay after treatment with 31.2 nM Taxol at different time-points. **C**, Expression of LC3B-I and LC3B-II in MCF-7 cells was determined by western blotting after treatment with 31.2 nM Taxol at different time-points. **D**, p62 expression in MCF-7 cells was determined by western blotting after treatment with 31.2 nM Taxol for 24 h. Data are reported as means±SD of three independent experiments. *P<0.05, **P<0.01, *vs* control group (ANOVA).

**Figure 2.**
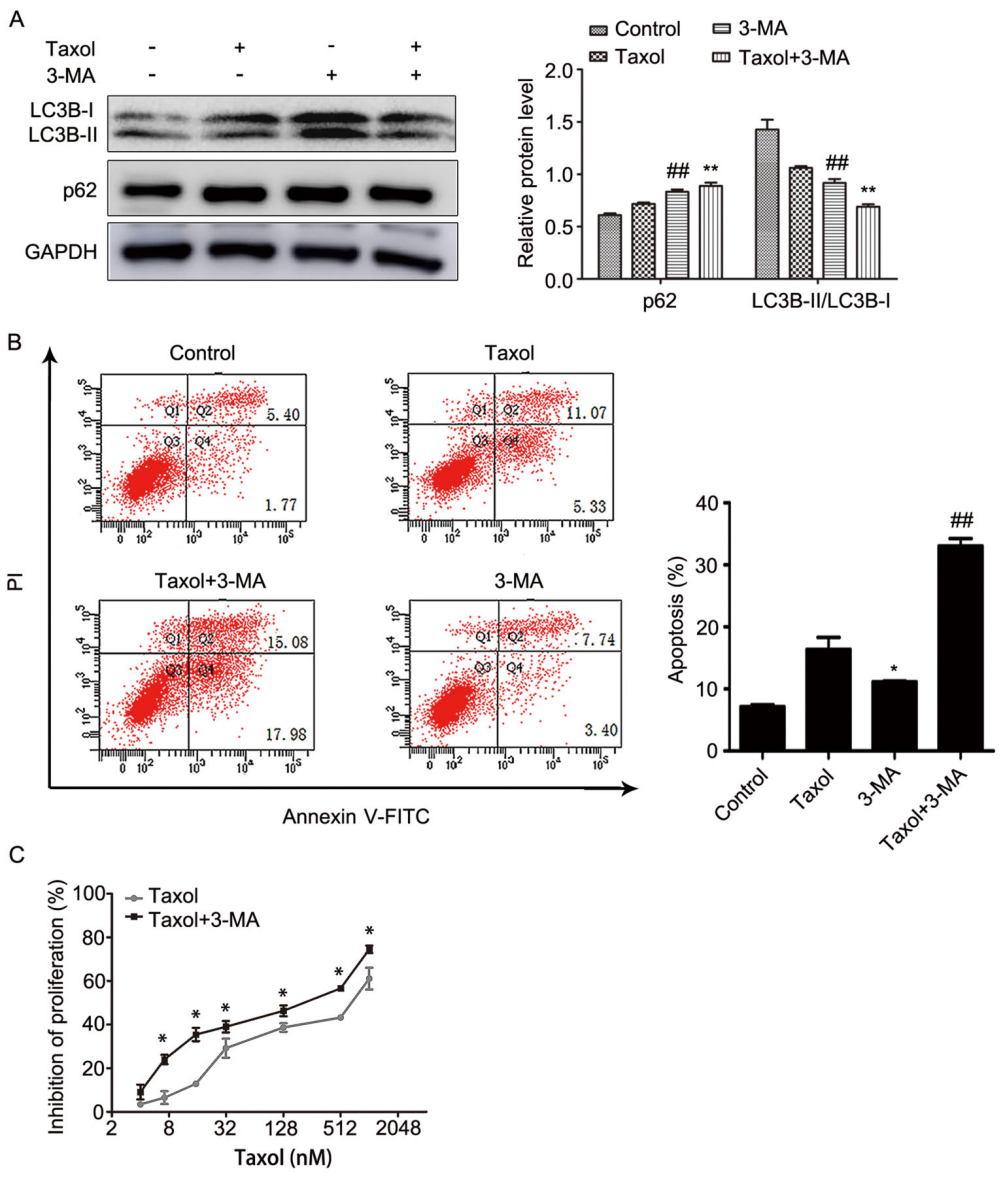
**A** and **B**, MCF-7 cells were treated with 5 mM 3-MA for 2 hours before 31.2 nM Taxol treatment for 24 h. LC3B-I, LC3B-II, and p62 expression in cells was determined by western blotting and cellular apoptosis was determined by flow cytometry. **C**, Cell proliferation was determined by the CCK-8 assay after pre-treatment with 5 mM 3-MA for 2 h and different concentrations of Taxol for 24 h. Data are reported as means±SD of three independent experiments. *P<0.05, **P<0.01, *vs* control group; ^##^P<0.01, *vs* Taxol group (ANOVA).

First, we used the CCK-8 assay to detect the inhibition of Taxol on MCF-7 cells proliferation. The results showed that IC_25_ of Taxol was approximately 31.2 nM at 24 h (Figure 1A). Then, we cultured MCF-7 cells for 24, 48, and 72 h in 31.2 nM Taxol. We found that the inhibition of Taxol on breast cancer MCF-7 cells depended on the time of Taxol treatment (Figure 1B). Last, we explored the relationship between autophagy and Taxol. LC3B was used as an important marker for autophagy. LC3B has two isoforms, LC3B-I and LC3B-II. When autophagy was up-regulated, LC3B-I was transformed into LC3B-II, and the ratio of LC3B-II/LC3B-I was increased. Therefore, the ratio of LC3B-II/LC3B-I can indicate the level of autophagy (34). As shown in (Figure 1C), the ratio of LC3B-II/LC3B-I decreased over time in 24 h after Taxol treatment. In addition, p62 can be degraded during the process of autophagy lysosome formation, thus the decrease in p62 usually signifies the occurrence of autophagy (35). Figure 1D showed that the expression of p62 was increased in Taxol-treated MCF-7 cells. These results showed Taxol exerted its cytotoxicity by inhibiting autophagy, and the inhibition of autophagy might increase the sensitivity of MCF-7 cells to Taxol. Thus, we used 3-MA, a selective PI3K inhibitor, to verify the above results. As shown in Figure 2A, the ratio of LC3B-II/LC3B-I was lowest and the expression of p62 was highest in the Taxol combined with 3-MA group. It indicated that 3-MA improved the inhibition of autophagy by Taxol. Furthermore, our data showed that the rate of apoptosis (Figure 2B) and the inhibition of cell proliferation (Figure 2C) by Taxol was increased in the Taxol-3-MA combined group. This indicated that the sensitivity of MCF-7 cells to Taxol and Taxol-induced cell apoptosis could be increased by inhibiting autophagy. Furthermore, these results suggested that the chemosensitivity of MCF-7 cells to Taxol can be improved by inhibiting autophagy and inducing apoptosis.

### miR-129-5p enhanced chemosensitivity of Taxol by inhibiting autophagy and promoting apoptosis in MCF-7 cells

**Figure 3.**
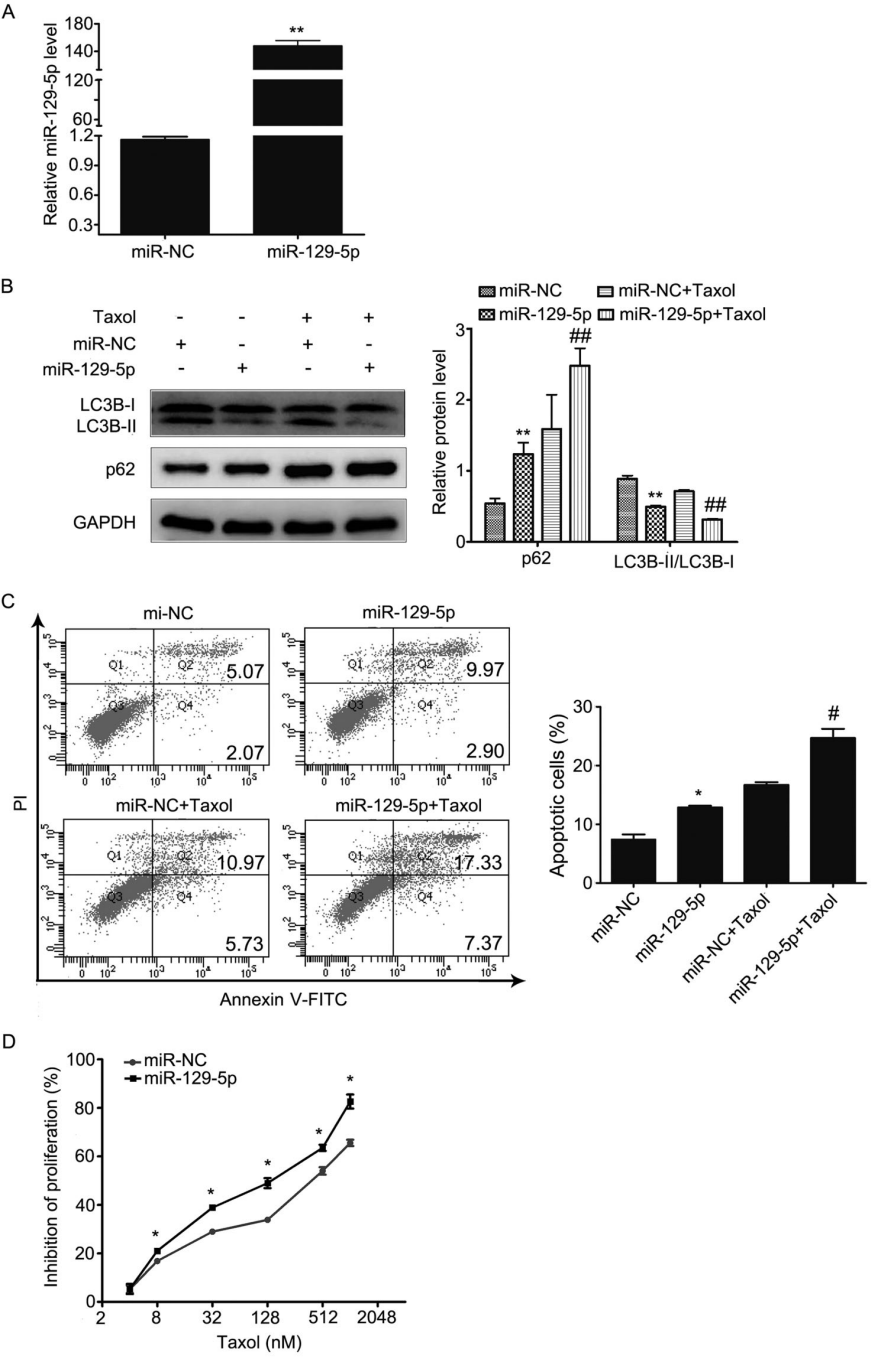
**A**, Relative miR-129-5p expression was detected by qRT-PCR analysis in MCF-7 cells transfected with miR-129-5p mimics or miR-NC. MiR-NC acted as a negative control. **B** and **C**, Cells were transfected with miR-NC or miR-129-5p mimics and then treated with 31.2 nM Taxol for 24 h. **B**, LC3B-I, LC3B-II, and p62 expression in MCF-7 cells were determined by western blot. **C**, Cellular apoptosis was determined by flow cytometry. **D**, Cell proliferation was determined by the CCK-8 assay after transfection with miR-NC or miR-129-5p mimics and treatment with different concentrations of Taxol for 24 h. Data are reported as the means±SD of three independent experiments. *P<0.05, **P<0.01, *vs* miR-NC group; ^#^P<0.05, ^##^P<0.01, *vs* miR-NC+Taxol group (ANOVA).

To explore whether miR-129-5p was involved in regulating the therapeutic effect of Taxol through the regulation of autophagy and apoptosis, we transfected miR-129-5p mimics into MCF-7 cells and then treated them with 31.2 nm of Taxol for 24 h. As shown in Figure 3A, miR-129-5p overexpression significantly increased the relative expression of miR-129-5p in MCF-7cells. Compared with miRNA-NC transfected cells, we found that miR-129-5p overexpression suppressed the conversion of LC3B-I to LC3B-II and inhibited the degradation of p62 with or without Taxol treatment (Figure 3B). This data strongly suggested that miR-129-5p could increase the inhibition of Taxol to autophagy. Then, we investigated whether miR-129-5p overexpression could enhance Taxol-induced apoptosis using flow cytometry. As shown in Figure 3C, miR-129-5p overexpression increased Taxol-induced apoptosis. Finally, we examined the effect of miR-129-5p in Taxol chemosensitivity using CCK-8 assays. Results showed that coupled with different concentrations of Taxol for 24 h, miR-129-5p overexpression significantly increased the inhibition of cell proliferation compared to the miR-NC group (Figure 3D). Taken together, these results support that miR-129-5p overexpression could increase the chemosensitivity of MCF-7 cells to Taxol by inhibiting autophagy and inducing apoptosis.

### HMGB1 was downregulated by miR-129-5p

To decipher the potential mechanisms promoting chemosensitivity in human MCF-7 cells by miR-129-5p, we used TargetScan, miRDB, and microRNA online analysis tools to search for the potential target genes of miR-129-5p. We found that there were eight overlapping target genes of miR-129-5p (Supplementary Figure S1A). Since HMGB1 is a unique regulator for autophagy among these eight overlapping target genes, we focused on researching HMGB1. The online database TargetScan indicated that there were two possible binding sites among miR-129-5p and HMGB1 (Supplementary Figure S1B). We also found that the expression of HMGB1 was higher in breast cancer tissue compared to normal breast tissue using the tumor database of Oncomine (Supplementary Figure S1C) and the Human Protein Atlas (Supplementary Figure S1D).

**Figure 4.**
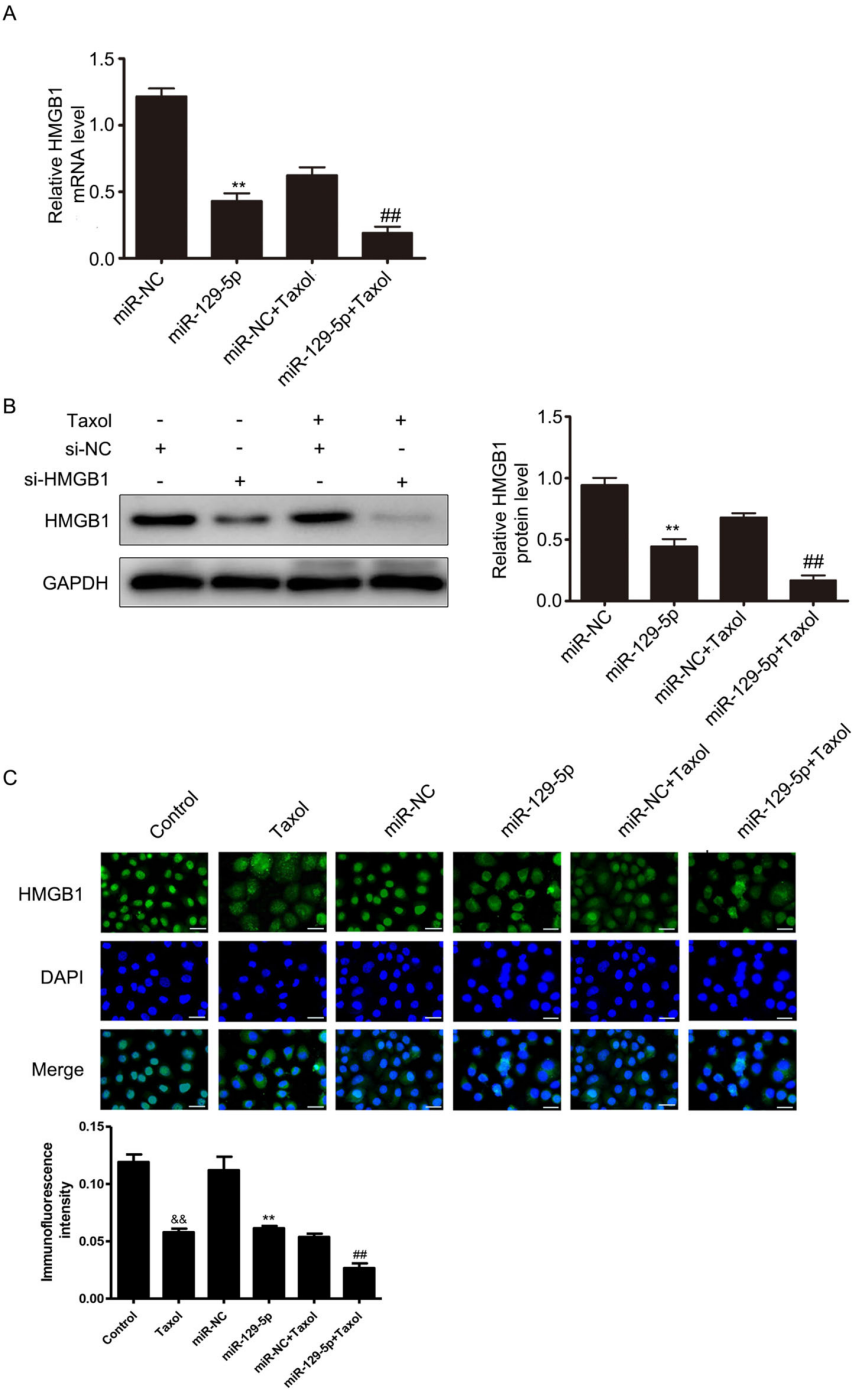
MiR-129-5p was negatively correlated with HMGB1 level. **A**, **B**, and **C**, Cells were transfected with miR-NC or miR-129-5p mimics, and then treated with 31.2 nM Taxol for 24 h. The levels of HMGB1 were determined by qRT-PCR (**A**), western blotting (**B**), and immunofluorescence assay (**C**). Scale bar, 100 μm. Data are reported as means±SD of three independent experiments. **P<0.01, *vs* miR-NC group; ^##^P<0.01, *vs* miR-NC+Taxol group; ^&&^P<0.01, *vs* control group (ANOVA).

To validate the effect of miR-129-5p on endogenous expression of HMGB1, we determined the levels of HMGB1 by qRT-PCR and western blotting in miR-129-5p transfected cells. Our results showed that miR-129-5p overexpression inhibited the expression of HMGB1 both at the mRNA (Figure 4A) and protein (Figure 4B) levels in MCF-7 cells. We also analyzed the inhibition effect of miR-129-5p on HMGB1 protein by immunofluorescence assay (Figure 4C). The results were consistent with Figure 4B, where the fluorescence intensity of HMGB1 was lowest in miR-129-5p transfected cells. These data indicated that miR-129-5p directly inhibited the endogenous expression of HMGB1 in MCF-7 cells.

### Downregulation of HMGB1 enhanced the chemosensitivity of Taxol by inhibiting autophagy and promoting apoptosis in MCF-7 cells

**Figure 5.**
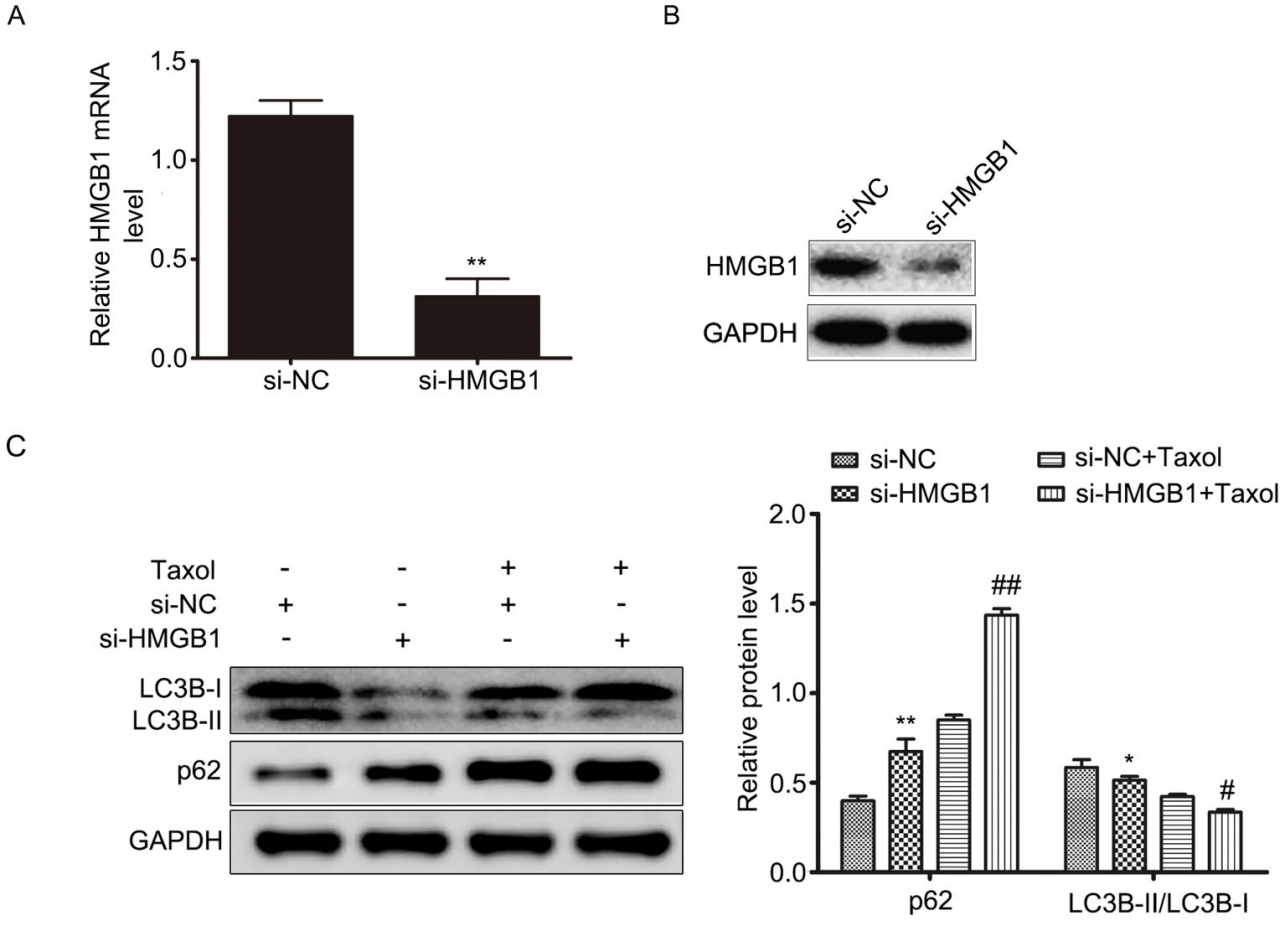
Interference of HMGB1 enhanced chemosensitivity of Taxol by inhibiting autophagy in MCF-7 cells. MCF-7 cells were transfected with si-HMGB1 or si-NC. **A**, Relative expression of HMGB1 mRNA was detected by qRT-PCR. **B**, Relative expression of HMGB1 protein was detected by western blot. **C**, MCF-7 cells were transfected with si-HMGB1 or si-NC and then treated with 31.2 nM Taxol for 24 h. LC3B-I, LC3B-II, and p62 expressions in cells were determined via western blot. Data are reported as means±SD of three independent experiments. *P<0.05, **P<0.01, *vs s*i-NC group; ^#^P<0.05, ^##^P<0.01, *vs* si-NC+Taxol group (ANOVA).

**Figure 6.**
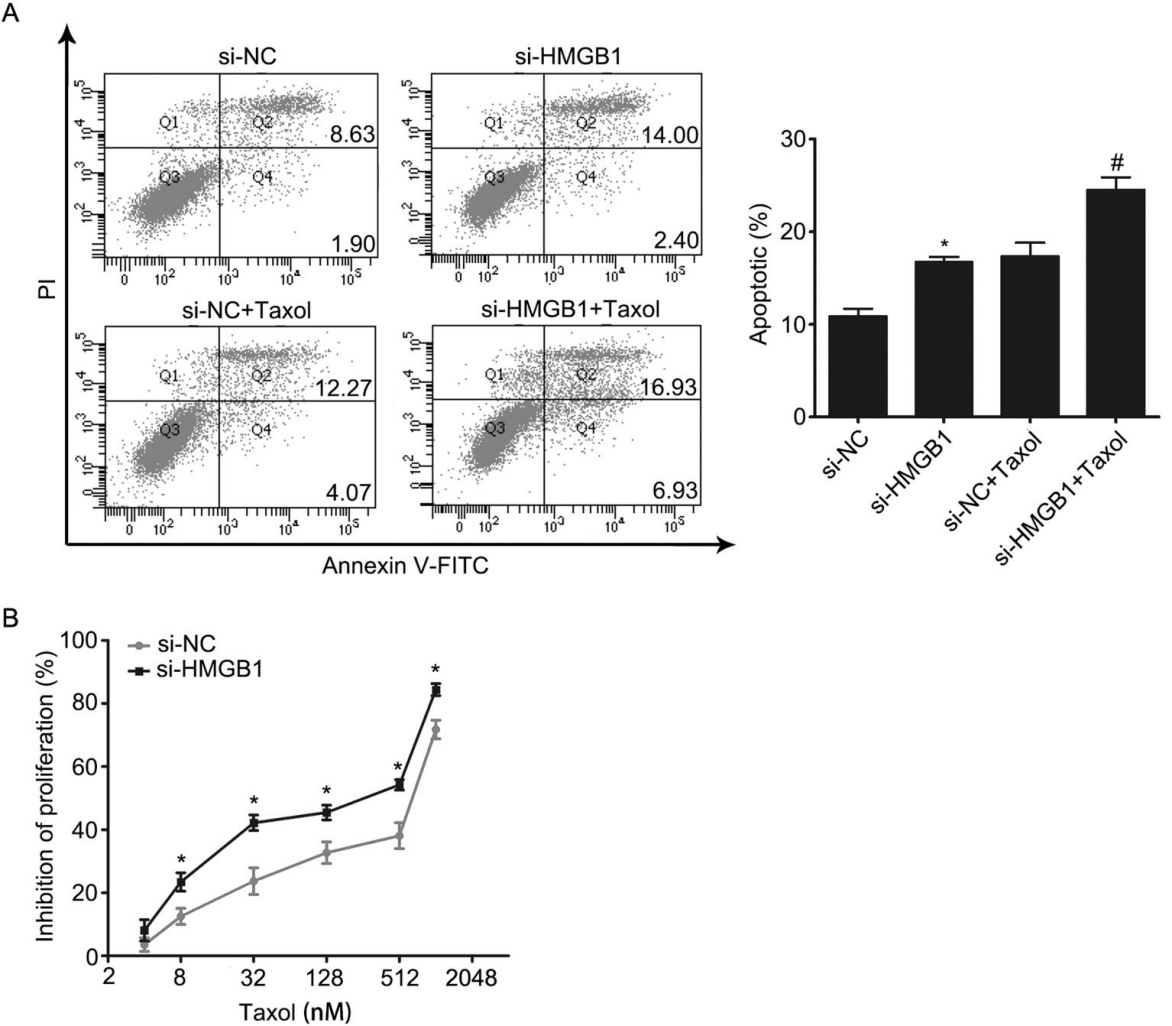
Interference of HMGB1 enhanced chemosensitivity of Taxol by promoting apoptosis in MCF-7 cells. **A**, Cellular apoptosis was determined by flow cytometry. The total rate of apoptotic cells is represented in a bar diagram from three independent experiments (right panel). **B**, Cells were transfected with si-NC or si-HMGB1, and then treated with different concentrations of Taxol for 24 h. Cell proliferation was determined by the CCK-8 assay. Data are reported as means±SD of three independent experiments. *P<0.05, *vs s*i-NC group; ^#^P<0.05, *vs* si-NC+Taxol group (ANOVA).

Based on the association of miR-129-5p and HMGB1, we hypothesized that miR-129-5p may inhibit autophagy and promote apoptosis through regulating HMGB1. We transfected si-HMGB1 or si-NC into MCF-7 cells and then treated with 31.2 nM Taxol to determine the function of HMGB1. First, our results showed that si-HMGB1 inhibited the expression of HMGB1 on both the mRNA (Figure 5A) and protein levels (Figure 5B). Subsequently, we detected the level of autophagy by western blotting. As shown in Figure 5C, the ratio of LC3B-II/LC3B-I was lowest in the transfected si-HMGB1 Taxol combined treatment group, but the expression of p62 was highest. These data indicated that downregulation of HMGB1 enhanced the inhibition of Taxol to autophagy. Furthermore, the results of flow cytometry demonstrated that downregulation of HMGB1 could increase Taxol-induced apoptosis (Figure 6A). Finally, the inhibition of cell proliferation was determined by CCK-8 after transfection with si-HMGB1 and exposure to different concentrations of Taxol for 24 h. The data demonstrated that si-HMGB1 increased the rate of Taxol-inhibited cell proliferation (Figure 6B). This suggested that the downregulation of HMGB1 could enhance the chemosensitivity of MCF-7 cells to Taxol by inhibiting autophagy and promoting apoptosis.

## Discussion

As an important chemotherapeutic agent for the treatment of cancer, including breast cancer, Taxol has successfully improved the survival of patients (7). However, some patients still have lower Taxol sensitivity because of drug resistance, which leads to poor prognosis of patients (8). Recently, altered levels of autophagy and miRNAs were attractive candidates for study as regulators of chemosensitivity in breast cancer (11,16,26). In the present study, we found that miR-129-5p increased the Taxol-induced apoptosis while inhibiting autophagy. Importantly, miR-129-5p increased the sensitivity of MCF-7 cells to Taxol by inhibiting autophagy. Further research demonstrated that miR-129-5p might inhibit autophagy by regulating HMGB1 and we confirmed this result in MCF-7 cells. Thus, we concluded that miR-129-5p increased the sensitivity of Taxol through inhibiting autophagy by regulating HMGB1. Furthermore, miR-129-5p/HMGB1/autophagy might be developed as a novel method for overcoming Taxol resistance in breast cancer.

Autophagy is a double-edged sword. Persistent and excessive autophagy can lead to tumor shrinkage and cell death. However, autophagy can protect cancer cells to maintain their survival in stress conditions (22,24). Recently, many studies have reported that autophagy was a significant factor in the development of drug resistance in breast cancer (25). In this study, we found that Taxol might exert its anti-cancer effect by inhibiting autophagy, implying that the level of autophagy might be associated with the sensitivity of Taxol. To validate this conclusion, we used 3-MA to inhibit autophagy. The results indicated that the inhibition of autophagy could increase the sensitivity of MCF-7 cells to Taxol and the level of apoptosis. These results suggested that autophagy had a pro-survival role in Taxol treatment of breast cancer cells. Meanwhile, autophagy had an anti-apoptosis effect.

To date, an increasing number of miRNAs have been identified to have a close relationship with the sensitivity of Taxol through modulating autophagy or apoptosis (36,37). Yet, the function of miR-129-5p in breast cancer and chemosensitivity is rarely reported. Recently, research demonstrated that miR-129-5p might play multiple and important roles in autophagy processes and drug resistance (38).

Generally, miRNAs play their biological role by targeting gene expression. We found HMGB1 was a target gene of miR-129-5p by searching online analysis tools and databases. Studies also have identified that miR-129-5p directly targets HMGB1 in breast cancer and osteosarcoma (33,39). Thus, it implies that miR-129-5p might enhance the sensitivity of Taxol by inhibiting HMGB1.

Recently, researchers reported that HMGB1 contributed to drug sensitivity of cancers by modulating autophagy and apoptosis (40). In our current study, we found HMGB1 was highly expressed in breast cancer by searching databases of Oncomine and the Human Protein Atlas. Many studies have also reported this result in breast cancers. Thus, our study explored the functions of HMGB1 on the sensitivity of Taxol. We found that changing the expression of HMGB1 could decrease the level of Taxol-mediated autophagy. More importantly, our data showed that downregulation of HMGB1 increased the Taxol-induced apoptosis and the sensitivity of MCF-7 cells to Taxol. These results indicated that inhibiting the expression of HMGB1 could improve the sensitivity of MCF-7 cells to Taxol by inhibiting autophagy and promoting apoptosis. However, this experiment used a single cell line, and such observations should be verified in a multi-cell line study design.

In conclusion, our study provides evidence that miR-129-5p overexpression increased the chemosensitivity of MCF-7 cells to Taxol through suppressing autophagy and promoting apoptosis by inhibiting HMGB1. This study may help to better understand the molecular mechanisms of drug resistance in breast cancer, and it may imply that overexpression of miR-129-5p alone or in conjunction with HMGB1 interference, is a promising strategy to combat multidrug resistance in breast cancer.

## Supplementary material

Click here to view [pdf].
